# Perioperative outcomes of robotic-assisted liver surgery: A comparison between patients with and without previous abdominal surgery

**DOI:** 10.1016/j.sopen.2026.06.002

**Published:** 2026-06-12

**Authors:** Anna Geiseler, Shadi Katou, Safa Öcal, Andreas Andreou, Felix Becker, Mazen Juratli, M. Haluk Morgül, Andreas Pascher, Benjamin Strücker

**Affiliations:** a Department of General, Visceral and Transplant Surgery, University Hospital Muenster, 48149, Muenster, Germany

**Keywords:** Robotic liver surgery, Minimally invasive surgery, Repeat hepatectomy, Robotic liver resection, Minimally invasive liver resection

## Abstract

**Background:**

Robotic-assisted liver surgery (RALSs) has numerous advantages over laparoscopic and open procedures. However, prior abdominal surgeries (PAS) are frequently considered as constraints for RALSs. Our study evaluated the impact of PAS on the intraoperative course and postoperative outcomes after RALSs.

**Materials and methods:**

For this retrospective cohort study, clinicopathological data were collected from all patients who underwent RALSs at the University Hospital Münster between December 2018 and March 2024. Patients were stratified based on whether they had undergone PAS or not (NPAS), the intraoperative course and postoperative outcomes after RALSs were analyzed.

**Results:**

In total, 116 patients were identified, 79 patients had undergone PAS, and 37 patients had not. The mean surgery time, conversion rate, intraoperative complications, postoperative intensive care unit (ICU) admissions and length of hospital stay (LOS) were comparable between the two groups. Forty-three patients had postoperative complications, without significant difference (PAS: 34 patients, NPAS: 9 patients). No deaths were observed within 30 days of surgery. One PAS patient died within 90 days of surgery.

**Discussion:**

Perioperative outcomes in patients undergoing RALSs were comparable in between PAS and NPAS patients and PAS was not associated with an increased risk of postoperative complications. PAS alone should not be considered as contraindication for RALSs.

## Highlights

• 68.1% of patients had a prior abdominal surgery.
• Patients with prior abdominal surgeries showed no prolonged surgery time.
• Conversion rate was not increased in patients with prior abdominal surgery.
• The occurrence of intra- and postoperative complications was comparable.
• Patients with prior abdominal surgeries did not show higher ICU admission rates.

## Graphical abstract

**Figure:**
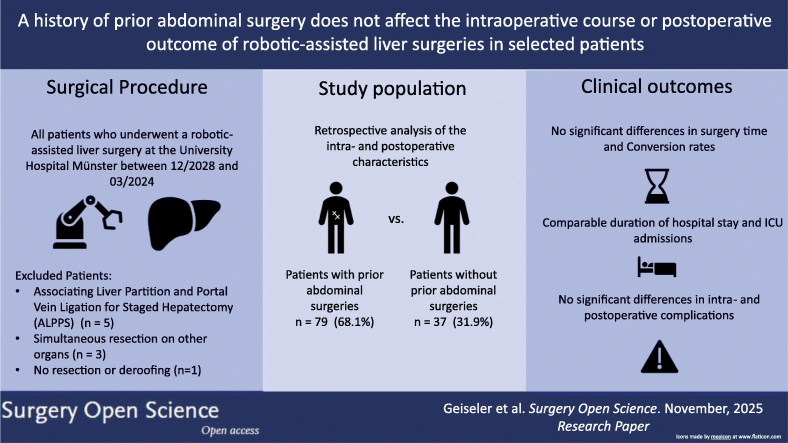

## Introduction

Compared with open surgical approaches, minimally invasive techniques have shown superiority for multiple indications in liver surgery. [1], [2], [3], [4] The advantages of laparoscopic techniques over open approaches include reduced blood loss, lower perioperative morbidity and shorter hospital stays while achieving comparable oncological results. [1], [2], [3], [4] Furthermore, laparoscopic procedures can enable a better re-operability for further surgeries, [2], [4] as their application can reduce the extent and severity of intraabdominal adhesions. [5], [6]

Another minimally invasive approach, which has gained popularity over the past few years, is the use of robotic platforms. [7], [8], [9] Providing three-dimensional vision, tremor filtration, and seven degrees of movement, robotic platforms can lead to higher precision and dexterity. [10], [11] Multiple studies have demonstrated that robotic platforms are safe and feasible to use in liver surgery, [7], [12], [13], [14], [15], [16] also on patients with reduced liver function. [14] Moreover, oncological efficiency can be achieved using robotic techniques. [7], [14] The use of robotic platforms can provide advantages over the laparoscopic approach, including reduced blood loss and lower conversion rates. [7], [13], [15] Compared to open approaches, robotic platforms can enable shorter hospital stays and also a reduced intraoperative blood loss. [7], [12], [14], [16]

A significant proportion of patients require multiple abdominal surgeries during their lifetime [17], consequently a history of abdominal surgery is an important aspect when choosing the appropriate surgical method. Historically, patients requiring liver surgery who had a history of prior abdominal surgeries (PAS) were assigned to open procedures. This decision stemmed from concerns about the increased surgical difficulty caused by adhesions, which frequently occur after abdominal surgeries, and their association with an increased risk for intraoperative complications, such as bowel injuries, and postoperative complications. [6], [18], [19]

With the increasing application of minimally invasive techniques, particularly robotics, we have expanded the indications for the robotic approach while achieving satisfactory results. At our highly specialized center for hepatobiliary surgery, the majority of liver surgeries are currently performed using robotic platforms, including in patients with a history of PAS. However, the available literature on robotic-assisted liver surgery (RALSs) after PAS and their impact on perioperative outcomes remains limited. To the best of our knowledge, only two studies have specifically addressed this topic. Thus, the primary objective of this study was to investigate the impact of PAS on the perioperative outcomes of RALSs and to supplement the existing literature with data from our high-volume tertiary center and teaching institution. We sought to contribute to a more comprehensive understanding of the role of PAS in the context of RALS. This study hypothesizes, that prior abdominal surgeries have no impact on the intraoperative course and postoperative outcome after RALS. Second, we investigated whether the type of PAS (e.g., open and major resections and procedures located in the upper abdomen) impact the outcome after RALSs.

## Materials and methods

### Study design and population

This study was designed as a retrospective cohort study. All consecutive patients who underwent RALSs, at the University Hospital in Münster between December 2018 and March 2024 were included. The University Hospital Münster is a high-volume tertiary center and teaching institution. All patients, procedures, and follow-up data were obtained from the routinely recorded clinical records of the University Hospital Münster, which are stored digitally in the hospital information system. A retrospective approach was chosen due to the availability of a well-maintained patient database, as well as to ensure that the analysis is based on real-world data. Approval for this study was given by the local ethics committee (ID: ID2019–636-f-S) on the 12th February 2021. Patients who underwent simultaneous surgery on other organs were excluded. The accepted concomitant procedures included cholecystectomy, lymphadenectomy (LAD), microwave ablation (MWA), local peritonectomy, and partial diaphragmatic resection. Patients who underwent an Associating Liver Partition and Portal Vein Ligation for Staged Hepatectomy (ALPPS) were also excluded. Patients were divided into two groups depending on whether they had previously undergone PAS or not (NPAS). Baseline characteristics and data on intraoperative course and postoperative outcomes were compared between these groups. PAS patients were further divided into three subgroups based on the procedure (open vs. minimally invasive), location (upper vs. lower abdomen), and extent (major vs. minor) of PAS. Major abdominal surgeries included resections of the stomach, liver, pancreas, and intestine, as well as transperitoneal nephrectomy, open cholecystectomy, peritonectomy, laparoscopic treatment of endometriosis in the upper abdomen, and open mesh insertion for median abdominal wall hernia. Minor abdominal surgeries included appendectomy, minimally invasive cholecystectomy, caesarean section, hysterectomy, adnexectomy, prostatovesiculectomy, inguinal hernia surgery, umbilical hernia surgery, and diagnostic laparoscopy. Patients who underwent laparoscopic and open abdominal surgery were assigned to the open PAS group. Patients who underwent surgery of the upper and lower abdomen were allocated to the upper abdominal PAS group.

### Collected data and definitions

Patients' baseline characteristics included age, sex, body mass index (BMI), American Society of Anesthesiologists (ASA) class, and Eastern Cooperative Oncology Group (ECOG) index. Data on smoking history, alcohol abuse and comorbidities including diabetes, cardiac disease, pulmonary disease, renal insufficiency, arterial hypertension, liver diseases, and liver cirrhosis were collected. Liver diseases include hepatitis B and C, cirrhosis, fibrosis, steatosis of the liver, and metabolic dysfunction-associated steatohepatitis. Further data concerning surgical indication, type and extent of liver surgery, and concomitant procedures were collected. Liver segments were defined according to Couinauds' segmental anatomical classification. [20] Liver resections were defined based on the Brisbane 2000 system of nomenclature of hepatic anatomy and resections. [21] In accordance with the Brisbane 2000 system, liver resections with ≤2 segments were defined as minor resections, and resections with ≥3 segments as major resections. [21] Patients who underwent a deroofing of hepatic cysts were assigned to the minor group. Perioperatively collected data included surgery time, conversion rate, intraoperative complications, intra- and postoperative transfusions of erythrocytes, intraoperative bowel injuries, length of hospital stay (LOS), intensive care unit (ICU) stay, reoperation, and hospital readmission within 30 days postoperatively. Surgery time was defined as the time from skin incision to closure. Postoperative complications were characterized according to the comprehensive complication index (CCI) and Clavien-Dindo score (CD). [22] Complications with a CD ≤ 3a are regarded as “mild” whereas complications ≥3b are considered “severe”. Furthermore, data on non-surgical complications were collected. These included pneumonias, pulmonary embolisms, STEMIs/non-STEMIs, urinary tract infections, sepsis, unexplained increases in infection values, electrolyte disorders, elevated lactate levels, elevated transaminase levels, hypertensive crises, pleural effusions, pneumothoraces, delirium/ hepatic encephalopathies, complications due to positioning (hematomas, nerve injuries), intestinal paralysis, edema, thromboses, renal insufficiencies/ acute kidney injuries, panaritiums and cardiac complications except STEMI & Non-STEMI & and hypertensive crises. 30 days morbidity and mortality, as well as 90 days mortality were assessed.

### Surgical procedure

Until 2021 minor liver resections and deroofings of hepatic cysts were performed using the da Vinci Surgical X System™ (Intuitive Surgical, Inc. 1020 Kifer Road, Sunnyvale, CA 94086–5304, USA). Since 2021 all RALSs were performed using the da Vinci Surgical Xi System™ (Intuitive Surgical, Inc. 1020 Kifer Road, Sunnyvale, CA 94086–5304, USA) including more complex RALSs, and a higher caseload.

A four-trocar approach with one assistance trocar was the standardized approach for each surgery. The liver parenchyma was resected using a combination of monopolar scissors, bipolar fenestrated forceps, and SynchroSeal (Intuitive Surgical, Inc. 1020 Kifer Road, Sunnyvale, CA 94086–5304, USA). Vascular control was achieved using Grena™ clips (Grena Limited,1000 Great West Road, Brentford, London, TW8 9HH, UK). For intraoperative sonographic control the BK5000 sonography unit and the Rob12C4 Robotic Transducer ultrasound probe (GE HealthCare, Mileparken 34, DK 2730 Herlev, Denmark) were applied combined with the TilePro technology (Intuitive Surgical, Inc. 1020 Kifer Road, Sunnyvale, CA 94086–5304, USA). The sonographic image is transmitted directly into the surgeon's field of vision. In some cases, the hepatoduodenal ligament was temporarily occluded using the Pringle maneuver. Indocyanine green was applied in individual cases to increase visualization of the tumor; it was administered intravenously the day or several days before surgery. In some cases, adhesiolysis was initiated laparoscopically using the Voyant Energy device (Applied Medical, 22872 Avenida Empresa, Rancho Santa Margarita, CA 92688, USA) until all robot trocars were placed, and adhesiolysis could be continued robotically. In some cases, additional trocars were placed for adhesiolysis.

### Statistical analysis

All statistical analyses were performed using IBM SPSS Statistics version 29.0.2.0 (IBM, Armonk, NY). Statistical significance was set at *p* < 0.05 (2-tailed). Categorical variables are expressed as numerical figures and percentages and were analyzed using the Fisher's exact test. Effect sizes were reported with odds ratios (OR) with 95% confidence intervals (CI) for binary outcomes and Cramér's V. Continuous variables were assumed not to be normally distributed and are expressed as medians and ranges. Data were analyzed using the Mann-Whitney *U* test and the Kruskal-Wallis test. Effect sizes for the Man-Whitney U test were calculated using the rank-biserial correlation (r) based on the standardized test statistic (Z) and total sample size (N). A multivariable logistic regression analysis was performed to assess independent associations between ASA class, BMI, Age and PAS and postoperative complications. The model included ASA class, Age, BMI and PAS as covariates. The results are presented with OR with 95% CI and *p*-Value (2-tailed).

## Results

### Population

During the study period, 125 patients underwent RALSs at the University Hospital Münster. Five patients who underwent an ALPPS, three patients who underwent a multivisceral resection and one patient who did not undergo resection or deroofing were excluded. Of the 116 remaining patients, 68.1% PAS patients (79/116) and 31.9% NPAS patients (37/116) were identified. Of the 79 PAS patients, 44.3% underwent laparoscopic (35/79) and 50.6% underwent open surgery (40/79); procedure data were missing for 5.1% of PAS patients (4/79). PAS was localized in the upper abdomen in 41.8% of PAS patients (33/79) and in the lower abdomen in 58.2% of PAS patients (46/79). 53.2% of PAS patients (42/79) underwent major surgery, and 46.8% of PAS patients (37/79) had minor PAS.

### Baseline characteristics

As Table 1 illustrates, baseline characteristics between PAS and NPAS patients were comparable regarding median age (60 years vs. 57 years, *p* = 0.387, *r* = 0.08), sex (*p* = 0.842, OR = 1.147, 95% CI 0.52–2.51), median BMI (25.5 vs. 26.2, *p* = 0.906, *r* = 0.01), ECOG (*p* = 0.841, Cramérs V = 0.096), history of smoking (*p* = 0.404, OR = 0.68, 95% CI 0.30–1.53), and alcohol abuse (*p* = 0.999, OR = 0.93, 95% CI 0.16–5.34). Significant differences exist in ASA class (*p* = 0.037, Cramér's V = 0.241), with a higher ASA class in PAS patients. Comorbidities such as diabetes (*p* = 0.418, OR = 1.78 95% CI 0.54–5.83), pulmonary disease (*p* = 0.765, OR = 0.82, 95% CI 0.26–2.65), cardiac disease (*p* = 0.446, OR = 1.76, 95% CI 0.60–5.20), renal insufficiency (*p* = 0.081, OR = 0.21, 95% CI 0.04–1.23), arterial hypertension (*p* = 0.689, OR = 0.80, 95% CI 0.37–1.75), liver disease (*p* = 0.794, OR = 0.84, 95% CI 0.31–2.33), and liver cirrhosis (*p* = 0.108, OR = 0.25, 95% CI 0.06–1.12) were comparable. Table 2 presents the surgical indications and the extent of surgery. The most frequent indications for RALSs in PAS patients were colorectal metastases (30.4%, 24/79), followed by hepatic cysts (17.7%, 14/79), and hepatocellular carcinoma (HCC) (11.4%, 9/79). For NPAS patients, the most common indications for RALSs were hepatic cysts (21.6%, 8/37), HCC (18.9%, 7/37), and cholangiocarcinoma (CCC) (16.2%, 6/37). There were significant differences in the surgical indication (*p* < 0.001). Liver metastases were the indication for surgery in 40.5% of PAS patients (32/79), while only 5.4% of NPAS patients (2/37) underwent surgery for this indication (p < 0.001). 40.5% of NPAS patients (15/37) and 21.6% of PAS patients (17/79) underwent surgery due to primary hepatobiliary malignancies (*p* = 0.045). Benign lesions were the surgical indication in 38% of PAS patients (30/79) and in 54.1% of NPAS patients (20/37) (*p* = 0.113). The extent (*p* = 0.377) and type (*p* = 0.273) of liver surgery were comparable in both groups. Concomitant procedures were performed in 39.2% of PAS patients (31/79) and in 64.9% of NPAS patients (24/37) (*p* = 0.016).

**Table 1:** Baseline characteristics of patients.

Parameter	Total *n* = 116	PAS *n* = 79	NPAS *n* = 37	p – Value	Effect size
Age (median and range)	59.50 (21–86)	60.0 (21–86)	57.0 (21–84)	0.387^a^	U: 1315.5 Z: - 0.865 r: 0.08
Sex (n, %)				0.842^b^	OR: 1.147 95% CI: 0.52–2.51
Male	56 (48.3)	39 (49.4)	17 (45.9)		
Female	60 (51.7)	40 (50.6)	20 (54.1)		
BMI (kg / m^2^, median and range)	25.5 (17.3–46.4)	25.5 (17.3–46.4)	26.2 (17.9–42.9)	0.906^a^	U: 1441.5 Z: −0.118 r: 0.01
ASA classification (n, %)				0.037^b^	Cramér's V: 0.241
1	7 (6.0)	3 (3.8)	4 (10.8)		
2	66 (56.9)	41 (51.9)	25 (67.6)		
3	34 (29.3)	28 (35.4)	6 (16.2)		
Missing	9 (7.8)	7 (8.9)	2 (5.4)		
ECOG (n, %)				0.841^b^	Cramér's V: 0.096
0	55 (47.4)	36 (45.6)	19 (51.4)		
1	45 (38.8)	32 (40.5)	13 (35.1)		
2	8 (6.9)	6 (7.6)	2 (5.4)		
3	1 (0.9)	1 (1.3)	0 (0.0)		
4	0 (0.0)	0 (0.0)	0 (0.0)		
5	0 (0.0)	0 (0.0)	0 (0.0)		
Missing	7 (6.0)	4 (5.1)	3 (8.1)		
Diabetes mellitus (n, %)	18 (84.5)	14 (17.7)	4 (10.8)	0.418^b^	OR: 1.78 95% CI: 0.54–5.83
Pulmonary disease (n, %)	14 (12.1)	9 (11.4)	5 (13.5)	0.765^b^	OR: 0.82 95% CI: 0.26–2.65
Cardiac disease (n, %)	22 (19.0)	17 (21.5)	5 (13.5)	0.446^b^	OR: 1.76 95% CI: 0.60–5.20
Renal insufficiency (n, %)	6 (5.2)	2 (2.5)	4 (10.8)	0.081^b^	OR: 0.21 95% CI: 0.04–1.23
Arterial hypertension (n, %)	52 (44.8)	34 (43.0)	18 (48.6)	0.689^b^	OR: 0.80 95% CI: 0.37–1.75
Liver disease (n, %)	20 (17.2)	13 (16.5)	7 (18.9)	0.794^b^	OR: 0.84 95% CI: 0.31–2.33
Liver cirrhosis (n, %)	8 (6.9)	3 (3.8)	5 (13.5)	0.108^b^	OR: 0.25 95% CI: 0.06–1.12
Smoking history (n, %)	40 (34.5)	25 (31.6)	15 (40.5)	0.404^b^	OR: 0.68 95% CI: 0.30–1.53
Alcohol abuse (n, %)	6 (5.2)	4 (5.1)	2 (5.4)	0.999^b^	OR: 0.93 95% CI: 0.16–5.34

OR, Odds Ratio; CI, Confidence Interval; BMI, Body Mass Index; ASA, American Society of Anesthesiologists; ECOG, Eastern Cooperative Oncology Group.

a : Mann-Whitney-*U* Test.

b : Fisher's exact test.

**Table 2:** Indication for robotic-assisted liver surgery and extent of surgery.

Parameter	Total n = 116	PAS *n* = 79	NPAS *n* = 37	P-value
Indication (n, %)				<0.001^a^
Primary hepatobiliary malignancy	32 (27.6)	17 (21.6)	15 (40.5)	0.045^a^
HCC	16 (13.8)	9 (11.4)	7 (18.9)	
CCC	9 (7.8)	3 (3.8)	6 (16.2)	
Gallbladder carcinoma	5 (4.3)	4 (5.1)	1 (2.7)	
HCC & CCC	1 (0.9)	1 (1.3)	0 (0.0)	
Lymphoma	1 (0.9)	0 (0.0)	1 (2.7)	
Metastatic disease	34 (29.3)	32 (40.5)	2 (5.4)	< 0.001 a
CRLM	24 (20.7)	24 (30.4)	0 (0.0)	
Other	10 (8.6)	8 (10.0)	2 (5.4)	
Benign	50 (43.1)	30 (38.0)	20 (54.1)	0.113 a
FNH	10 (8.6)	5 (6.3)	5 (13.5)	
Hepatic adenoma	5 (4.3)	4 (5.1)	1 (2.7)	
Cyst	22 (19.0)	14 (17.7)	8 (21.6)	
Hemangioma	8 (6.9)	5 (6.3)	3 (8.1)	
Echinococcus cyst	4 (3.4)	1 (1.3)	3 (8.1)	
Abscess	1 (0.9)	1 (1.3)	0 (0.0)	
Liver resection (n, %)				0.273^a^
Hemihepatectomy right	5 (4.3)	3 (3.8)	2 (5.4)	
Hemihepatectomy left	3 (2.6)	1 (1.3)	2 (5.4)	
Right lateral	1 (0.9)	1 (1.3)	0 (0.0)	
Left lateral	18 (15.5)	11 (13.9)	7 (18.9)	
Atypical resection	48 (41.4)	38 (48.1)	10 (27.0)	
Segment resection	22 (19.0)	12 (15.2)	10 (27.0)	
Deroofing	18 (15.5)	12 (15.2)	6 (16.2)	
Pericystectomy	1 (0.9)	1 (1.3)	0 (0.0)	
Extend of liver resection (n, %)				0.377 a
Minor	83 (71.6)	54 (68.4)	29 (78.4)	
Major	33 (28.4)	25 (31.6)	8 (21.6)	
Concomitant procedure (n, %)	55 (47.4)	31 (39.2)	24 (64.9)	0.016 a
Cholecystectomy	40 (34.5)	23 (29.1)	17 (45.9)	
LAD	8 (6.9)	4 (5.1)	4 (10.8)	
Microwave ablation	3 (2.6)	3 (3.8)	0 (0.0)	
Local peritonectomy	1 (0.9)	0 (0.0)	1 (2.7)	
Cholecystectomy and LAD	2 (1.7)	1 (1.3)	1 (2.7)	
Cholecystectomy, LAD and partial diaphragmatic resection	1 (0.9)	0 (0.0)	1 (2.7)	

HCC, hepatocellular carcinoma; CCC, cholangiocarcinoma; CRLM, colorectal liver metastasis; FNH, focal nodular hyperplasia; LAD, lymphadenectomy.

a : Fisher's exact test.

### Intra-and postoperative characteristics

Table 3, Table 4, Table 5 show the intra- and postoperative characteristics of the PAS and NPAS patients. The mean surgery time (234 vs. 247 min, *p* = 0.799) did not differ significantly. Conversions to open surgery were required in 3.8% of PAS patients (3/79) and 2.7% of NPAS patients (1/37), without significant difference (*p* = 0.999). No conversion to open surgery was caused by adhesions. In two PAS patients, conversion was performed controlled due to the tumor location and an increased bleeding tendency. The conversions in one PAS patient and in one NPAS patient were emergencies caused by bleeding and respiratory insufficiency, respectively. Intraoperative complications occurred in 5.1% of PAS patients (4/79) and 2.7% of NPAS patients (1/37), (*p* = 0.999). Intraoperative transfusions of red blood cells were required in 3.8% of PAS patients (3/79) and 2.7% of NPAS patients (1/37) (p = 0.999). Intraoperative bowel injuries occurred in 2.5% of PAS patients (2/79) and 0.0% of NPAS patients (0/37) (p = 0.999), they could be directly linked to conversions but had no further consequences after intraoperative repair. Postoperative transfers to the ICU occurred in 19% of PAS patients (15/79) and 16.2% of NPAS patients (6/37) (*p* = 0.801), with a mean stay of two days (range 1–13 days) (*p* = 0.621). Readmissions to the ICU after the transfer to a normal ward were required in 2.5% of PAS patients (2/79) and 0.0% of NPAS patients (0/37) (*p* = 0.999). Postoperative transfusions of red blood cells were required in 1.3% of PAS patients and 2.7% of NPAS patients (1/37) (*p* = 0.319). Overall, postoperative complications occurred in 37.1% of patients (43/116) within 30 days of surgery, with 43% of PAS patients (34/79) and 24.3% of NPAS patients (9/37), (*p* = 0.064). “Severe” complications occurred in 3.8% of PAS patients (3/79) and 2.7% of NPAS patients (1/37) whereas “mild” complications occurred in 39.2% of PAS patients (31/79) and 21.6% of NPAS patients (8/37) (*p* = 0.133). Bile leaks occurred in 5.1% of PAS patients (4/79) and 5.4% of NPAS patients (2/37) (*p* = 0.999). Abdominal bleeding occurred in 1.3% of PAS patients (1/79) and 5.4% of NPAS patients (2/37) (*p* = 0.238). Wound infections occurred in 1.3% of PAS patients (1/79) and 1.3% of NPAS patients (1/37) (*p* = 0.538). Only non-surgical complications showed significant difference, occurring in 39.2% of PAS patients (31/79) and 18.9% of NPAS patients (7/37) (*p* = 0.035). Individual non-surgical complications are presented in Table 5 with no statistically significant differences observed for any single complication. The LOS was not significantly different (*p* = 0.561). Readmissions to hospital after discharge occurred in 5,1% of PAS patients (4/79) and in 2.7% of NPAS patients (1/37), without significant difference (p = 0.999). 90-days mortality was 1.3% in PAS patients (1/79) and 0% (0/37) in NPAS patients (p = 0.999).

**Table 3:** Intraoperative characteristics.

	Total n = 116	PAS n = 79	NPAS n = 37	*p*-Value
Conversion to open surgery (n, %)	4 (3.4)	3 (3.8)	1 (2.7)	0.999^a^
Surgery time (median and range)	241.5 (26–725)	234.0 (51–671)	247.00 (26–725)	0.799^b^
Intraoperative complications (n, %)	5 (4.3)	4 (5.1)	1 (2.7)	0.999 a
Intraoperative transfusion (n, %)	4 (3.4)	3 (3.8)	1 (2.7)	0.999^a^
Intraoperative bowel injury (n, %)	2 (1.7)	2 (2.5)	0 (0.0)	0.999^a^

a : Fisher's exact test.

b : Mann-Whitney-U Test.

**Table 4:** Postoperative characteristics.

	Total *n* = 116	PAS n = 79	NPAS n = 37	*p*-Value
Length of hospital stay in days (median and range)	6.00 (2–37)	6.0 (2–37)	6.0 (3–27)	0.561^a^
ICU stay (yes) (n, %)	21 (18.1)	15 (19.0)	6 (16.2)	0.801^b^
Duration of ICU stay in days (median and range)	2 (1−13)	2 (1–13)	2 (1–3)	0.621^a^
ICU readmission (n, %)	2 (1.7)	2 (2.5)	0 (0.0)	0.999^b^
Postoperative transfusion (n, %)	2 (1.7)	1 (1.3)	1 (2.7)	0.319^b^
Reoperation (n, %)	4 (3.4)	3 (3.8)	1 (2.7)	0.999^b^
Postoperative complications (n, %)	43 (37.1)	34 (43.0)	9 (24.3)	0.064^b^
Clavien Dindo Score (n, %)				0.263^b^
1	18 (15.5)	16 (20.3)	2 (5.4)	
2	9 (7.8)	7 (8.9)	2 (5.4)	
3a	12 (10.3)	8 (10.1)	4 (10.8)	
3b	3 (2.6)	2 (2.5)	1 (2.7)	
4	0 (0.0)	-0 (0.0)	-0 (0.0)	
5	1 (1.3)	1 (1.3)	-0 (0.0)	
Grouped Clavien Dindo Score (n, %)				0.133
Mild (Grade 1, 2 & 3a)	39 (33.6)	31 (39.2)	8 (21.6)	
Severe (3b, 4 & 5)	4 (3.4)	3 (3.8)	1 (2.7)	
CCI (median and range)	20.9 (8.7–100)	20.9 (8.7–100.0)	26.2 (8.7–50.7)	0.241^a^
Readmission to hospital (n, %)	5 (4.3)	4 (5.1)	1 (2.7)	0.999^b^
Bile leak (n, %)	6 (5.2)	4 (5.1)	2 (5.4)	0.999^b^
Abscess (n, %)	0 (0.0)	0 (0.0)	0 (0.0)	–
Abdominal bleeding (n, %)	3 (2.6)	1 (1.3)	2 (5.4)	0.238^b^
Wound infection (n, %)	2 (1.7)	1 (1.3)	1 (2.7)	0.538^b^
30 days morbidity (n, %)				0.064^b^
Complications	43 (37.1)	34 (43.0)	9 (24.3)	
No complications	73 (62.9)	45 (57.0)	28 (75.7)	
30 days mortality (n, %)				–
Alive	116 (100.0)	79 (100.0)	37 (100.0)	
Dead	0 (0.0)	0 (0.0)	0 (0.0)	
90 days mortality (n, %)				0.999^b^
Alive	115 (99.1)	78 (98.7)	37 (100.0)	
Dead	1 (0.9)	1 (1.3)	0 (0.0)	

ICU, Intensive care unit; CCI, comprehensive complication index.

a : Mann-Whitney-U Test.

b : exact Fisher Test.

**Table 5:** Non-surgical complications.

	Total	PAS	NPAS	p-Value
Non-surgical complications	38 (32.8)	31 (39.2)	7 (18.9)	0.035^a^
Pneumomia	4 (3.4)	3 (3.8)	1 (2.7)	0.999 a
Pulmonary embolism	2 (1.7)	2 (2.5)	0 (0.0)	0.999 a
STEMI / Non-STEMI	0 (0.0)	0 (0.0)	0 (0.0)	–
Urinary tract infection	7 (6.0)	6 (7.6)	1 (2.7)	0.428 a
Sepsis	3 (2.6)	2 (2.5)	1 (2.7)	0.999 a
Unexplained increase in infection values	2 (1.7)	2 (2.5)	0 (0.0)	0.999 a
Electrolyte disorder	7 (6.0)	5 (6.3)	2 (5.4)	0.999 a
Elevated lactate level	1 (0.9)	1 (1.3)	0 (0.0)	0.999 a
Elevated transaminase level	1 (0.9)	1 (1.3)	0 (0.0)	0.999 a
Hypertensive crisis	1 (0.9)	1 (1.3)	0 (0.0)	0.999 a
Pleural effusion	10 (8.6)	8 (10.1)	2 (5.4)	0.499 a
Pneumothorax	2 (1.7)	2 (2.5)	0 (0.0)	0.999 a
Delirium / hepatic encephalopathy	2 (1.7)	2 (2.5)	0 (0.0)	0.999 a
Due to positioning (hematoma, nerve injury)	2 (1.7)	2 (2.5)	0 (0.0)	0.999 a
Intestinal paralysis	1 (0.9)	1 (1.3)	0 (0.0)	0.999 a
Urinary retention	1 (0.9)	1 (1.3)	0 (0.0)	0.999 a
Edema	2 (1.7)	2 (2.5)	0 (0.0)	0.999 a
Exanthema	1 (0.9)	1 (1.3)	0 (0.0)	0.999 a
Thrombosis	1 (0.9)	1 (1.3)	0 (0.0)	0.999 a
Renal insufficiency / acute kidney injury	5 (4.3)	3 (3.8)	2 (5.4)	0.653 a
Panaritium	1 (0.9)	0 (0.0)	1 (2.7)	0.319 a
Cardiac complications exept STEMI & Non-STEMI & and hypertensive crises	2 (1.7)	2 (2.5)	0 (0.0)	0.999 a

a : Fisher's exact test.

The results of the multivariable logistic regression analysis are shown in Table 6. ASA class (OR = 0.82, 95% CI 0.37–1.85, *p* = 0.639), PAS (OR = 2.15, 95% CI 0.83–5.54, *p* = 0.114), and age (OR = 1.03, 95% CI 1.00–1.07, *p* = 0.053) were not independently associated with postoperative complications. BMI was significantly associated with postoperative complications (OR = 1.13, 95% CI 1.03–1.23, *p* = 0.012).

**Table 6:** Multivariable logistic regression analysis for postoperative complications.

Variable	OR (95% CI)	p-Value
ASA class	0.82 (0.37–1.85)	0.639
Age (years)	1.03 (1.00–1.07)	0.053
BMI (kg / m^2^)	1.13 (1.03–1.23)	0.012
Prior abdominal surgery	2.15 (0.83–5.54)	0.114

OR, Odds Ratio; CI, Confidence Interval; ASA, American Society of Anesthesiologists; BMI, Body Mass Index.

The results of subgroup analyses are presented in Table 7. The intra- and postoperative characteristics showed no significant deviations, and the occurrence of nonsurgical complications was also found to be statistically non-significant.

**Table 7:** Intra- and postoperative characteristics of the subgroup-analysis.

	Total	NPAS	PAS: Open vs. laparoscopic			PAS: Upper Abdomen vs. lower Abdomen			PAS: Major vs. Minor		
			Open *n* = 40	laparoscopic *n* = 35	p-Value	Upper Abdomen *n* = 33	Lower Abdomen *n* = 46	p-Value	Major *n* = 42	Minor n = 37	p-Value
Conversion to open (n,%)	4 (3.4)	1 (2.7)	1 (2.5)	2 (2.57)	0.687^a^	2 (6.1)	1 (2.2)	0.682^a^	2 (4.8)	1 (2.7)	0.999^a^
Length of operation in minutes (median and range)	241.5 (26–725)	247.0 (26–725)	227.0 (59–630)	264.0 (51–671)	0.763^b^	242.0 (59–671)	219.5 (51–630)	0.923^b^	255.0 (67–671)	219.00 (51–630)	0.373^b^
Intraoperative complications (n, %)	5 (4.3)	1 (2.7)	3 (7.5)	0 (0.0)	0.323^a^	0 (0.0)	4 (8.7)	0.221^a^	1 (2.4)	3 (8.1)	0.521^a^
Intraoperative transfusion (n, %)	4 (3.4)	1 (2.7)	1 (2.5)	2 (5.7)	0.687^a^	2 (6.1)	1 (2.2)	0.682^a^	2 (4.8)	1 (2.7)	0.999^a^
Bowel injury (n,%)	2 (2.7)	0 (0.0)	2 (5.0)	0 (0.0)	0.328^a^	0 (0.0)	2 (4.3)	0.334^a^	1 (2.4)	1 (2.7)	0.999^a^
Duration of hospital stay in days (median and range)	6.00 (2–37)	6.00 (3–27)	6 (3–37)	6 (2−21)	0.745^b^	6 (2–37)	6 (3–37)	0.836^b^	6 (2–37)	6 (2–37)	0.844^b^
ICU stay (yes) (n,%)	21(18.1)	6 (16.2)	9 (22.5)	5 (14.3)	0.644^a^	8 (24.2)	7 (15.2)	0.593^a^	8 (19.0)	3 (8.1)	0.956^a^
Duration of ICU stay in days (median and range)	2 (1–13)	2 (1–3)	2 (2–4)	2 (1–13)	0.553^b^	2,5 (2−13)	2 (1–3)	0.428^b^	2 (1–4)	2 (1–13)	0.885^b^
ICU readmission (n,%)	2 (1.7)	0 (0.0)	1 (2.5)	1 (2.9)	0.762^a^	0 (0.0)	2 (4.3)	0.334^a^	0 (0.0)	2 ((5.4)	0.200^a^
Postoperative transfusion (n,%)	2 (1.7)	1 (2.7)	1 (2.5)	0 (0.0)	0.999^a^	0 (0.0)	1 (2.2)	0.999^a^	0 (0.0)	1 (2.7)	0.534^a^
Reoperation (n,%)	4 (3.4)	1 (2.7)	2 (5.0)	1 (2.9)	0.999^a^	0 (0.0)	3 (6.5)	0.461^a^	1 (2.4)	2 (5.4)	0.835^a^
Postoperative complications (n,%)	43 (37.1)	9 (24.3)	18 (45.0)	15 (42.9) 1	0.131^a^	14 (42.4)	20 (43.5)	0.154^a^	20 (47.6)	14 (37.8)	0.112^a^
Clavien Dindo Score (n,%)					0.290 a			0.229^a^			0.243^a^
1	18 (15.5)	2 (5.4)	8 (20.0)	7 (20.0)		7 (21.2)	9 (19.6)		11 (26.2)	5 (13.5)	
2	9 (7.8)	2 (5.4)	2 (5.0)	5 (14.3)		5 (15.2)	2 (4.3)		5 (11.9)	2 (5.4)	
3a	12 (10.3)	4 (10.8)	6 (15.0)	2 (5.7)		2 (6.1)	6 (13.0)		3 (7.1)	5 (13.5)	
3b	3 (2.6)	1 (2.7)	1 (2.5)	1 (2.9)		0 (0.0)	2 (4.3)		1 (2.4)	1 (2.7)	
4	0 (0.0)	0 (0.0)	0 (0.0)	0 (0.0)		0 (0.0)	0 (0.0)		0 (0.0)	0 (0.0)	
5	1 (0.9)	0 (0.0)	1 (2.5)	0 (0.0)		0 (0.0)	1 (2.2)		0 (0.0)	1 (2.7)	
Grouped Clavien Dindo Score (n,%)					0.294^a^			0.163^a^			0.181^a^
Mild (≤3a)	39 (33.6)	7 (18.9)	16 (40.0)	14 (40.0)		14 (42.4)	17 (37.0)		19 (45.2)	12 (32.4)	
Severe (≥3b)	4 (3.4)	1 (2.7)	2 (5.0)	1 (2.9)		0 0 (0.0)	3 (6.5)		1 (2.4)	2 (5.4)	
CCI (median and range)	20.9 (8.7–100.0)	26.2 (8.7–50.7)	21.75 (8.7–100)	20.90 (8.7–48.2)	0.219^b^	20.9 (8.7–62.2)	21.75 (8.7–100.0)	0.263^b^	16.550 (8.7–62.2)	29.85 (8.7–100.0)	0.255^b^
Readmission to hospital (n,%)	5 (4.3)	1 (2.7)	3 (7.5)	1 (2.9)	0.618^a^	0 (0.0)	4 (8.7)	0.221^a^	1 (2.4)	3 (8.1)	0.521^a^
Bile leak (n,%)	6 (5.2)	2 (5.4)	3 (7.5)	1 (2.9)	0.871^a^	2 (6.1)	2 (4.3)	0.999^a^	2 (4.8)	2 (5.4)	0.999^a^
Abscess (n,%)	0 (0.0)	0 (0.0)	0 (0.0)	0 (0.0)	–	0 (0.0)	0 (0.0)	–	0 (0.0)	0 (0.0)	–
Abdominal bleeding (n,%)	3 (2.6)	2 (5.4)	1 (2.5)	0 (0.0)	0.526^a^	0 (0.0)	1 (2.2)	0.493^a^	0 (0.0)	1 (2.7)	0.301^a^
Wound infection (n,%)	2 (1.7)	1 (2.7)	1 (2.5)	0 (0.0)	0.999^a^	0 (0.0)	1 (2.2)	0.999^a^	0 (0.0)	1 (2.7)	0.534^a^
Non-surgical complications (n,%)	38 (32.8)	7 (18.9)	16 (40.0)	14 (40.0)	0.082^a^	13 (39.4)	18 (39.1)	0.093^a^	18 (42.9)	13 (35.1)	0.064 a
Pneumonia (n,%)	4 (3.4)	1 2.7)	1 (2.5)	2 (5.7)	0.687^a^	3 (9.1)	0 (0.0)	0.066^a^	2 (4.8)	1 (2.7)	0.999^a^
Pulmonary embolism (n,%)	2 (1.7)	0 (0.0)	1 (2.5)	1 (2.9)	0.762^a^	0 (0.0)	1 (4.3)	0.334^a^	0 (0.0)	2 (5.4)	0.200^a^
STEMI / NSTEMI (n,%)	0 (0.0)	0 (0.0)	0 (0.0)	0 (0.0)	–	0 (0.0)	0 (0.0)	–	0 (0.0)	0 (0.0)	–
Urinary tract infection (n,%)	7 (6.0)	1 (2.7)	3 (7.5)	3 (8.6)	0.624^a^	3 (9.1)	3 (6.5)	0.485^a^	4 (9.5)	2 (5.4)	0.558 a
Sepsis (n,%)	3 (2.6)	1 (2.7)	1 (2.5)	1 (2.9)	0.999^a^	1 (3.0)	1 (2.2)	0.999^a^	1 (2.4)	1 (2.7)	0.999^a^
30 days morbidity (n,%)					0.131^a^			0.154^a^			0.112^a^
Complications	43 (37.1)	9 (24.3)	18 (45.0)	15 (42.9)		14 (42.4)	20 (43.5)		20 47.6)	14 (37.8)	
No complications	73 (62.9)	28 (75.7)	22 (55.0)	20 (57.1)		19 (57.6)	26 (56.5)		22 (52.4)	23 (62.2)	
30 days mortality (n,%)					–			–			–
Alive	116 (100.0)	37 (100.0)	40 (100.0)	35 (100.0)		33 (100.0)	46 (100.0)		42 (100.0)	37 (100.0)	
Dead	0 (0.0)	0 (0.0)	0 (0.0)	0 (0.0)		0 (0.0)	0 (0.0)		0 (0.0)	0 (0.0)	
90 days mortality (n,%)					0.999^a^			0.999^a^			0.638^a^
Alive	115 (99.1)	37 (100.0)	39 (97.5)	35 (100.0)		33 (100.0)	45 (97.8)		42 (100.0)	36 (97.3)	
Dead	1 (0.9)	0 (0.0)	1 (2.5)	0 (0.0)		0 (0.0)	1 (2.2)		0 (0.0)	1 (2.7)	

ICU, Intensive care unit; CCI, comprehensive complication index.

a : Fishers exact test.

b : Kruskal-Wallis-Test.

## Discussion

This study aimed to investigate the impact of PAS on the intraoperative course and postoperative outcomes of RALSs. Regarding the baseline characteristics, PAS patients had significantly higher ASA classes compared to NPAS patients (*p* = 0.035) with a small to moderate association (Cramér's V = 0.24), suggesting a higher perioperative risk in this group. Other baseline characteristics showed no significant differences. Overall, intra- and postoperative characteristics between PAS and NPAS patients were largely comparable. However, the overall incidence of non-surgical complications was significantly higher in PAS patients (p = 0.035). When individual non-surgical complications were analyzed separately, no significant differences were observed between the groups. In the multivariable logistic regression analysis, neither the ASA class (OR = 0.82, 95% CI 0.37–1.85, *p* = 0.639), nor PAS (OR = 2.15, 95% CI 0.83–5.54, *p* = 0.114), nor age (OR = 1.03, 95% CI 1.00–1.07, *p* = 0.053) were independently associated with postoperative complications. BMI was significantly associated with postoperative complications (OR = 1.13, 95% CI 1.03–1.23, *p* = 0.012), indicating that higher BMI may increase the risk of postoperative complications.

Our findings support the results of previous studies that demonstrated the feasibility of RALSs in patients with PAS. A prospective observational study by Feldbrügge et al. showed no significant differences between the occurrence of postoperative complications in patients who underwent RALSs depending on their history of PAS. Furthermore, they showed that a history of PAS, including prior liver surgeries, is not an independent risk factor for a prolongation in surgery time. Thus, they concluded that robotic assisted liver resections could be safely performed in patients with PAS. [23] Another study by Sucandy et al. showed no significant differences in the perioperative outcomes, including surgery time, intraoperative estimated blood loss, the occurrence of intra- and postoperative complications, 30 days mortality and readmissions to hospital after surgery, of patients who underwent robotic-assisted hepatectomy based on their history of prior liver surgeries, PAS, and those without. Interestingly, they observed no intraoperative complications and no postoperative ICU admissions at all. Their conclusion was that robotic-assisted repeat hepatectomies can be performed safely while achieving perioperative outcomes comparable to robotic-assisted primary hepatectomies. [24] Vancoillie et al. compared robotic-assisted with laparoscopic repeat liver resections and showed that robotic-assisted repeat liver resections are safe to perform and can even provide advantageous short-term outcomes such as shorter duration of hospital stay. [25]

PAS frequently leads to intra-abdominal adhesions. [5], [18] These may adversely affect further abdominal surgeries and their perioperative outcomes, being linked to a prolongation of surgery time and LOS. [18], [19] Moreover, adhesions can increase the incidence of intraoperative complications and the postoperative morbidity. [18], [19] Bowel injuries in particular are a frequent and feared complication. [18] Our analysis showed no differences between PAS and NPAS patients in terms of surgery time, intraoperative complications, and bowel injuries. The two intraoperative bowel injuries in our cohort both occurred in PAS patients, they were treated intraoperatively and did not lead to a significant prolongation in the operation time or further complications. Furthermore, LOS and overall postoperative morbidity and mortality did not differ between the groups.

Some authors have concluded that adhesions are associated with an increased risk of conversion. [26] For example, Cipriana et al. showed that patients receiving laparoscopic liver resections had an increased risk for conversion when they had received previous surgeries. [27] Interestingly, in our cohort, we did not observe differences in the conversion rate between patients with PAS and NPAS, which is in accordance with the results of Feldbrügge et al. and Sucandy et al. [23], [24]

None of the conversion in our cohort were attributed to adhesions and no adhesiolysis was performed in these patients. Instead, in two PAS patients, a controlled conversion was required due to tumor location and an increased bleeding tendency. Furthermore, emergency conversions were required in one PAS patient due to bleeding and in one NPAS patient due to respiratory insufficiency. However, adhesions were not systematically assessed in our cohort. Therefore, although no conversion was directly attributed to adhesions, their potential indirect impact on intraoperative decision-making cannot be excluded. This may partly explain the discrepancy between our finding and previous reports identifying adhesions as risk factor for conversions. [26], [27] Another explanation for the relatively low conversion rate may be the usage of the robotic platform. An international multicenter study from Saleh et al. showed that the use of robotic platforms can lower the risk for conversions significantly. [26] Similar results were obtained in a multicenter study by Milone et al. stating that robotic-assisted surgery can lower the risk of adhesiolysis-related conversions in hepatectomies compared to laparoscopic hepatectomies. [28] In addition, the increasing surgical experience with laparoscopic and robotic platforms may have contributed to these findings. The severity of adhesions correlates with the procedure and the extent of prior abdominal surgery. Consequently, open and major resections are linked to more severe adhesions than minor and laparoscopic surgeries. [5] Furthermore, adhesions are more likely to develop in areas of PAS. Therefore, it is reasonable to assume that patients who undergo open or major surgeries, as well as those located in the upper abdomen, may face a higher risk when undergoing subsequent liver resections. We found no significant differences in perioperative outcomes between NPAS patients and those who underwent minimally invasive surgeries, minor surgeries, or surgeries in the lower abdomen.

Our study has several limitations. One limiting factor is the retrospective study design, inherently carrying the risk of selection bias and confounding. Another limitation is the uneven distribution in size between PAS and NPAS patients, combined with partly small sample sizes. Both factors can increase variability, reduce statistical power and introduce bias. To address potential confounders a multivariable logistic regression analysis was performed. Residual confounding cannot be excluded. Furthermore, the unequal group distribution reflects real-world clinical practice, which can enhance the external validity of the results. In addition, the study includes early cases from the implementation phase of robotic liver surgery at our center. Therefore, a potential learning curve effect cannot be excluded, which may have influenced operative performance and perioperative outcomes.

In conclusion, our results show that PAS did not adversely affect the intraoperative course or postoperative outcomes of RALSs. No significant differences were observed between PAS and NPAS patients regarding surgery time, conversion rate, incidence of intraoperative complications, postoperative morbidity, or mortality. Furthermore, PAS was not independently associated with an increased risk of postoperative complications. These findings indicate that RALSs in PAS patients may result in comparable perioperative outcomes to those of NPAS patients. However, these results should be interpreted with caution given the retrospective design, limited sample size and the uneven distribution between PAS and NPAS groups. This study does not allow conclusions regarding patient selection or the identification of high-risk subgroups among PAS patients.

Based on our institutional experience, RALSs can be successfully performed in PAS patients, and these patients may benefit from advantages of a robotic-assisted approach. Therefore, PAS alone should not be considered a contraindication for RALSs, and a robotic approach may be considered in carefully evaluated patients rather than being generally avoided in PAS patients.

## CRediT authorship contribution statement

**Anna Geiseler:** Writing – review & editing, Writing – original draft, Validation, Project administration, Methodology, Investigation, Formal analysis, Data curation. **Shadi Katou:** Investigation, Data curation. **Safa Öcal:** Data curation. **Andreas Andreou:** Investigation. **Felix Becker:** Writing – review & editing, Investigation. **Mazen Juratli:** Investigation. **M. Haluk Morgül:** Writing – review & editing, Investigation. **Andreas Pascher:** Supervision, Resources, Conceptualization. **Benjamin Strücker:** Writing – review & editing, Validation, Supervision, Project administration, Methodology, Investigation, Formal analysis, Conceptualization.

## Author contributions

All authors have contributed substantially to the interpretation of data, participation in drafting the article and revising it critically for important intellectual content and gave their final approval of the manuscript before submission.

## Informed consent statement

Informed consent was obtained from all subjects involved in this study.

## Institutional review board statement

This study was performed in line with the principles of the Declaration of Helsinki. Approval was granted by the Ethics Committee of the University of Muenster (Muenster, Germany) and the Medical Association of Westphalia-Lippe (ID: ID2019–636-f-S) on 12 February 2021.

## Declaration of Generative AI and AI-assisted technologies in the writing process

During the preparation of this work the authors used DeepL in order to translate terms from German to English. After using this tool, the authors reviewed and edited the content as needed and take full responsibility for the content of the publication.

During the preparation of this work the authors used ChatGPT by OpenAI in order to find synonyms and improve readability. After using this tool, the authors reviewed and edited the content as needed and take full responsibility for the content of the publication.

## Funding statement

This research did not receive any specific grant from funding agencies in the public, commercial, or not-for-profit sectors.

## Declaration of competing interest

The authors declare that they have no known competing financial interests or personal relationships that could have appeared to influence the work reported in this paper.

## Data Availability

The data presented in this study are available on request from the corresponding author. The data are not publicly available.

## Glossary

ALPPS: Associating Liver Partition and Portal Vein Ligation for Staged Hepatectomy
ASA: American Society of Anesthesiologists
BMI: Body mass index
CCC: Cholangiocarcinoma
CCI: Comprehensive complication index
CI: Confidence Interval
CD: Clavien-Dindo score
ECOG: Eastern Cooperative Oncology Group
HCC: Hepatocellular carcinoma
ICU: Intensive care unit
LAD: Lymphadenectomy
LOS: Length of hospital stay
MWA: Microwave ablation
NPAS: No prior abdominal surgery
OR: Odds Ratio
PAS: Prior abdominal surgery
RALSs: Robotic-assisted liver surgery
